# Clinical Characteristics and Management of Drug-Induced Lupus in Patients Receiving Tumor Necrosis Factor Inhibitor Therapy

**DOI:** 10.7759/cureus.72388

**Published:** 2024-10-25

**Authors:** Ali Kimiaei, Seyedehtina Safaei, Malik Kasapoglu, Fulya Coşan

**Affiliations:** 1 Internal Medicine, Bahçeşehir University, Istanbul, TUR

**Keywords:** case series, dil, drug-induced lupus, sle, tnf inhibitors

## Abstract

Introduction

Drug-induced lupus (DIL) is an autoimmune condition triggered by exposure to certain medications, leading to the emergence of clinical features that resemble those of systemic lupus erythematosus (SLE). This study aims to investigate the clinical characteristics, management strategies, and outcomes of patients who develop DIL during tumor necrosis factor inhibitor (TNFi) therapy.

Methods

We conducted a retrospective case series involving 15 patients who developed positive anti-double-stranded DNA (anti-dsDNA) antibodies while undergoing TNFi therapy between 2015 and 2023. Clinical data were collected and analyzed to assess the relationship between TNFi therapy and the onset of DIL symptoms.

Results

The case series included 15 patients (13 females and two males) with a mean age of 42.13 years. The primary diagnoses were ankylosing spondylitis (AS) (46.66%), rheumatoid arthritis (RA) (40%), and psoriatic arthritis (PsA) (13.33%). The mean duration from TNFi initiation to the detection of anti-dsDNA positivity was 5.13 years. Two patients (13.33%) exhibited clinical manifestations of DIL; however, no major organ involvement was observed. Sixty percent of patients continued with the same TNFi without disease activation, while 33% switched medications.

Conclusion

TNFi-induced DIL typically presents with mild symptoms. Both continuation and switching of TNFi therapy can be considered without significant exacerbation of symptoms. Management should be individualized based on clinical and laboratory findings. Further research with larger patient cohorts is warranted to determine optimal management strategies.

## Introduction

Drug-induced lupus (DIL) is an autoimmune condition in which exposure to certain drugs leads to the development of clinical characteristics similar to those of systemic lupus erythematosus (SLE) in some aspects [1]. Although there are no established international diagnostic criteria for DIL, the commonly used parameters for diagnosis include the use of one or more drugs known to be associated with this condition for at least one month, the development of at least one clinical feature characteristic of SLE, and the resolution of symptoms upon withdrawal of the medications [1,2].

While numerous drugs have been implicated in DIL, this discussion will specifically focus on tumor necrosis factor inhibitors (TNFi), which are frequently used in rheumatology and often prescribed for patients with high disease activity. Data on the follow-up and treatment of DIL associated with TNFi are limited and primarily derived from case reports, suggesting that while cases have been reported, comprehensive studies are lacking. These reported cases often result in the discontinuation of the implicated drug; however, in some situations, limited alternative treatment options make discontinuation impractical or necessitate the reintroduction of the therapy after cessation. Here, we present the clinical characteristics and follow-up data of patients who developed DIL during treatment with TNF inhibitors.

The findings of this article were previously accepted as an abstract for publication at the European Alliance of Associations for Rheumatology (EULAR) 2024 Congress.

## Materials and methods

Study design and patient selection

This retrospective study was conducted at Bahçeşehir University Hospital Medical Park Göztepe between 2015 and 2023. We included 15 patients diagnosed with DIL secondary to TNFi therapy. Patients were selected based on the development of anti-double-stranded DNA (anti-dsDNA) antibodies during their follow-up for various rheumatologic conditions, including ankylosing spondylitis (AS), rheumatoid arthritis (RA), and psoriatic arthritis (PsA). Data were collected from electronic medical records, including clinical, serological, and demographic information.

Inclusion and exclusion criteria

Patients were included if they had been on TNFi therapy, exhibited positive anti-dsDNA antibodies, and presented with at least one symptom suggestive of lupus, such as fatigue, photosensitivity, or a malar rash. Patients were excluded if they had previously been diagnosed with SLE or if they had discontinued TNFi therapy for reasons unrelated to DIL.

Criteria for requesting anti-dsDNA testing

Anti-dsDNA testing was requested for patients exhibiting one or more of the following symptoms: a positive antinuclear antibody (ANA) test, photosensitivity, malar rash, fatigue, unexplained weight loss, constitutional symptoms (fever, malaise), hair loss, or elevated acute-phase reactants (such as C-reactive protein or erythrocyte sedimentation rate). These symptoms, combined with TNFi use, raised suspicion of DIL, even in the absence of classic SLE criteria.

Data collection

Clinical data were collected retrospectively, including age, sex, underlying diagnosis, and the duration of TNFi therapy before anti-dsDNA positivity. Serological parameters, including anti-dsDNA levels, ANA titers, and extractable nuclear antigen (ENA) profiles, were evaluated. The clinical manifestations of DIL, such as rash, fatigue, and joint pain, were documented during routine follow-up visits.

Ethical approval

Due to the case series nature of this study, institutional review board (IRB) approval was waived in accordance with our institution's policy. However, informed consent was obtained from all patients prior to their participation in the study.

## Results

Fifteen patients were included in this study, with a mean age of 42.13 years; the group comprised two males (13.33%) and 13 females (86.67%). Diagnoses included seven cases of AS (46.67%), six cases of RA (40%), and two cases of PsA (13.33%), with an average disease duration of eight years. The mean duration from the initiation of the first TNFi therapy to the onset of positive anti-dsDNA was 5.13 years, with a median of four years (range, two to 14 years). Among the 15 patients, nine received adalimumab (60%), three received certolizumab (20%), two received infliximab (13.33%), and one received golimumab (6.67%). Six patients (40%) had a history of previous TNFi use, with exposure ranging from one to four different TNFi, while nine patients (60%) had not used any TNFi before. The average duration between the first TNFi use and the detection of anti-dsDNA positivity was 41.53 months, with a median of 24 months (range, 3-156 months). The average duration between the last TNFi use and the detection of anti-dsDNA positivity was 21.8 months, with a median of 12 months (range, three to 92 months).

Throughout the follow-up period after the onset of anti-dsDNA positivity (mean 14.06 months, median eight months, range one to 56 months), anti-dsDNA levels remained stable in six patients (40%) and became negative in nine patients (60%). Notably, two patients (13.33%) exhibited clinical manifestations of DIL, with one presenting with malar rash, lupus dermatitis, and arthritis, and the other displaying only a malar rash. No major organ involvement was observed in any of the patients. ANA titers were negative in three patients (20%) and positive in 12 patients (80%), with three of them exhibiting high titers. Seven patients (46.67%) tested positive for ENAs. Patient-specific details are presented in Table 1.

**Table 1:** Patient demographics and clinical findings M: male; F: female; AS: ankylosing spondylitis; RA: rheumatoid arthritis; PSA: psoriatic arthritis; TNFi: tumor necrosis factor inhibitor; anti-dsDNA: anti-double-stranded DNA antibodies; ANA: antinuclear antibodies; ENA: extractable nuclear antigen; AMA-M2: anti-mitochondrial antibody M2; anti-RNP: anti-ribonucleoprotein antibodies; -: negative; +: positive

Patient	Age/gender	Diagnosis	Disease duration (months)	Years post-TNFi initiation when anti-dsDNA tested positive	TNFi during DNA positivity	Follow-up period after anti-dsDNA positivity (months)	Course of anti-dsDNA	Clinical presentation	ANA	ENA	Treatment choice
1	45/M	AS	8	5	Certolizumab	18	Same level	Asymptomatic	-	-	Changed to etanercept
2	26/F	AS	9	8	Certolizumab	20	-	Malar rash, lupus dermatitis, arthritis	+	Anti-histone, anti-nucleosome, anti-Sm antibodies	Changed to infliximab
3	60/F	AS	16	14	Infliximab	1	-	Asymptomatic	+ (High titer)	-	Continuation of the same medication
4	44/F	AS	4	3	Adalimumab	3	Same level	Asymptomatic	+ (High titer)	-	Continuation of the same medication
5	31/F	RA	6	2	Adalimumab	8	-	Asymptomatic	-	-	Continuation of the same medication
6	23/F	RA	9	2	Certolizumab	6	Same level	Asymptomatic	+	Anti-histone, anti-nucleosome	Changed to adalimumab
7	36/F	PSA	5	4	Adalimumab	32	-	Asymptomatic	+ (High titer)	anti-RNP, anti-mitochondrial M2 antibody (AMA-M2), anti-Ku antibodies, anti-nucleosome	Changed to baricitinib
8	35/F	RA	4	2	Adalimumab	16	-	Asymptomatic	-	Anti-DFS70	Changed to rituximab
9	48/F	AS	6	5	Golimumab	24	-	Asymptomatic	+	-	Continuation of the same medication
10	58/F	RA	7	4	Infliximab	3	-	Malar rash	+	Anti-nucleosome, AMA-M2	Continuation of the same medication
11	45/M	AS	13	2	Adalimumab	6	Same level	Asymptomatic	+ (High titer)	-	Changed to etanercept
12	38/F	RA	8	7	Adalimumab	56	-	Asymptomatic	+ (High titer)	Anti-Sm antibodies, Anti-RNP antibody	Continuation of the same medication
13	48/F	RA	9	8	Adalimumab	9	Same level	Asymptomatic	+	-	Continuation of the same medication
14	48/F	AS	9	8	Adalimumab	6	Same level	Asymptomatic	+	-	Continuation of the same medication
15	47/F	PSA	7	3	Adalimumab	3	-	Asymptomatic	-	Anti-Ro antibody	Continuation of the same medication

During follow-up, hydroxychloroquine was administered to all patients with DIL, and close monitoring was maintained as the patients exhibited very mild symptoms. Sixty percent of patients (n=9) continued the same TNFi without experiencing disease activation. In 33% of patients (n=5), a medication switch was performed due to the activation of the primary disease. In one case of RA (6%), the drug was changed to rituximab. In patients who switched to a second TNFi, anti-dsDNA positivity persisted; however, no flares, major organ involvement, or new lupus development were observed during the follow-up period.

## Discussion

DIL associated with TNFi therapy is a recognized phenomenon in clinical practice. Our case series highlights several key features observed in patients who developed DIL following TNFi therapy. The principal characteristics of these cases have been organized and discussed within distinct sections, including clinical features, treatment modalities, and outcomes. The cases presented in this series demonstrate the variability in the presentation and course of TNFi-induced lupus.

Pre-existing disease

In our case series, we observed a distribution of rheumatological conditions that differed from what is typically reported in the literature. While RA is the most commonly reported condition in relation to TNFi-induced lupus, our case series showed a different pattern [3]. The majority of our cases were AS, constituting 46.67% of the patients, followed by RA at 40% and PsA representing 13.33%.

The predominance of AS cases in our study is particularly noteworthy. Given that AS is not an autoimmune disease like RA and autoantibody formation is infrequent in AS, the presence of ds-DNA positivity in these patients is more likely to be associated with TNFi therapy.

This distribution pattern in our case series suggests important clinical implications. For AS patients, the limited alternative treatment options often contribute to difficulties in discontinuing TNFi medication. Unlike in RA, transitioning to B-cell therapies is not feasible for AS patients, often necessitating the continuation of the drug or a switch to another TNFi.

These observations from our case series highlight the need for careful monitoring and management of TNFi therapy in AS patients, given their potentially higher risk of developing DIL and the challenges in treatment modification. The contrast between our findings and typical literature reports emphasizes the importance of considering DIL in AS patients on TNFi therapy, despite it being less commonly reported than in RA.

Clinical manifestations and major organ involvement

Typically, DIL is associated with mild clinical symptoms, as indicated by several studies that have demonstrated only mucocutaneous and articular manifestations [1]. However, in some cases, there is evidence of major organ involvement, such as the kidneys, central nervous system (CNS), and heart [2,4-6]. In our study, two patients showed signs of DIL, with one presenting with malar rash, lupus dermatitis, and arthritis, and the other displaying only a malar rash; however, no major organ involvement was observed. Our study also revealed the absence of significant major organ involvement, with only mild clinical findings.

Autoantibodies

The high frequency of autoantibody induction, including ANA and anti-dsDNA antibodies, in patients treated with TNFi agents is well documented [7,8]. The administration of TNF-α antagonists causes elevated titers of ANAs in patients who already started treatment with positive ANA serology, while new-onset positive ANA may also develop in previously negative ANA patients who are treated with TNF-α inhibitors [7]. Other antibodies, like anti-histone antibodies, anticardiolipin antibodies, and anti-Smith antibodies, have also been reported [7,9,10]. Positive ENAs may also develop in patients on anti-TNF-α agents [7]. In our cohort, anti-dsDNA was positive in all of our patients, ANA titers were negative in three patients (20%) and positive in 12 patients (80%), with three of them exhibiting high titers. Seven patients (46.67%) tested positive for ENAs (Table 1).

Responsible agents

Studies have estimated the incidence of DIL erythematosus to be 0.19% to 0.22% for infliximab, 0.18% for etanercept, and 0.10% for adalimumab during treatment [9,11-13]. In our patient group, nine patients (60%) received adalimumab, three (20%) received certolizumab, two (13.33%) received infliximab, and one (6.67%) received golimumab. However, as our study was not designed to investigate how many patients developed DIL for each TNFi specifically, we were unable to provide frequency data similar to those found in the literature.

As these biological agents have similar mechanisms of action, it is difficult to understand the differences in the risk of developing DIL due to each drug, but this difference may be attributed to variations in molecular structure, bioavailability, binding affinity, immunogenicity, and metabolism [14,15].

Management and prognosis

In managing DIL, the most common strategy reported in the literature is the discontinuation of TNFi, while a smaller proportion of cases involve switching to another TNFi [3-9,13,14]. In our cases, 60% of the patients continued the same TNFi without experiencing disease activation. A medication switch was performed in 33% of patients because of the activation of the primary disease. In one case with RA (6%), the drug was changed to rituximab. Regarding the prognosis of these patients, chronic SLE was observed in 8.0% (6 cases), significant improvement was noted following discontinuation of treatment in 81.3% (61 cases), improvement was observed after switching to another TNFi in 8.0% (six cases), and data regarding prognosis were not available in 2.7% (two cases). In our patients who switched to the second TNFi, anti-dsDNA positivity persisted; however, no flares, major organ involvement, or new lupus development were observed during the follow-up period.

This study has some limitations. First, the study only presented cases of DIL; thus, not all patients undergoing ANA testing in the outpatient clinic were screened. Second, there is ambiguity in defining patients with mild symptoms or dsDNA positivity as having DIL. The literature lacks clear consensus on this matter, which complicates interpretation and underscores the need for standardized definitions in future research. Additionally, this study was retrospective and had a small sample size. Moreover, treatment approaches lack standardized protocols, potentially affecting the consistency of management.

## Conclusions

In conclusion, particularly in conditions such as AS, where treatment modification options are limited, the potential continuation of ongoing therapies, including TNFi, may be necessary. In our study, we did not observe any recurrence of DIL symptoms when patients transitioned to another TNFi, nor did we note the progression or worsening of DIL symptoms when the same TNFi was continued. Given that the majority of patients in our cohort were asymptomatic despite anti-dsDNA positivity, we suggest that in the absence of significant clinical symptoms, continuing TNFi therapy with regular clinical and laboratory monitoring is a reasonable approach. Further research, including larger-scale studies, comprehensive follow-up, and standardized treatment protocols, is necessary for the effective management of DIL.
